# Do chiropractic college faculty understand informed consent: a pilot study

**DOI:** 10.1186/1746-1340-14-27

**Published:** 2006-12-21

**Authors:** Dana J Lawrence, Maria A Hondras

**Affiliations:** 1 Palmer Center for Chiropractic Research, 741 Brady Street, Davenport, IA 52803 USA

## Abstract

**Background:**

The purpose of this study was to survey full-time faculty at a single chiropractic college concerning their knowledge of Institutional Review Board (IRB) policies in their institution as they pertain to educational research.

**Methods:**

All full-time faculty were invited to participate in an anonymous survey. Four scenarios involving educational research were described and respondents were asked to select from three possible courses of action for each. In addition, respondents were queried about their knowledge of IRB policies, how they learned of these policies and about their years of service and departmental assignments.

**Results:**

The response rate was 55%. In no scenario did the level of correct answers by all respondents score higher than 41% and in most, the scores were closer to just under 1 in 3. Sixty-five percent of respondents indicated they were unsure whether Palmer had any policies in place at all, while 4% felt that no such policies were in place. Just over one-quarter (27%) were correct in noting that students can decline consent, while more than half (54%) did not know whether there were any procedures governing student consent.

**Conclusion:**

Palmer faculty have only modest understanding about institutional policies regarding the IRB and human subject research, especially pertaining to educational research. The institution needs to develop methods to provide knowledge and training to faculty. The results from this pilot study will be instrumental in developing better protocols for a study designed to survey the entire chiropractic academic community.

## Background

There is an expectation that faculty in higher education participate in scholarship and research in some fashion. In the current environment within the chiropractic academic community, there is also a growing emphasis on assessment and program development. Faculty members are expected to play a role in this process as well as share their experiences with each other and with the larger institutional and professional communities [1]. While not everyone has the skills or inclination to conduct health-care related research, those with primary assignments in instruction often find that their interests lead them to consider some form of educational research. Within a unionized environment, the possibility of conducting some form of research is one way to enhance the potential for advancement as well as fulfill hours of service required by the contract. At the same time, faculty members are not always clear about how to conduct research, nor are they aware of the requirements concerning when institutional review board (IRB) consideration and approval is needed. The situation is even more complicated when considering educational research. [2] Some misunderstand the implications of *45 CFR 46 *[3], the federal law governing human subjects research. *45 CFR 46 *states the following, in part:

EXEMPTION #1 (45 CFR 46.101(b)(1)):

*Research conducted in established or commonly accepted educational settings, involving normal educational practices, such as (i) research on regular and special education instructional strategies, or (ii) research on the effectiveness of or the comparison among instructional techniques, curricula, or classroom management methods*.

EXEMPTION #2 (45 CFR 46.101(b)(2)):

*Research involving the use of educational tests (cognitive, diagnostic, aptitude, achievement), survey procedures, interview procedures or observation of public behavior, unless: (i) information obtained is recorded in such a manner that human subjects can be identified, directly or through identifiers linked to the subjects; and (ii) any disclosure of the human subjects' responses outside the research that could reasonably place the subjects at risk of criminal or civil liability or be damaging to the subjects' financial standing, employability, or reputation*.

This is often interpreted by faculty to mean that no IRB involvement is necessary when conducting educational research. [4] This is complicated when information originally gathered for internal use only, such as for assessment purposes or accreditation activities, may lead to information that is of interest to a wider constituency, and therefore also lead to submission for a conference presentation or a manuscript. As Mavis and Henry noted [5], this blurs the distinction between evaluation (as specified exempt in *45 CFR 46*) and research.

While scientific research involving human subjects must by law and by necessity be submitted to the IRB after initial consideration by a Human Protections Administrator, the implications for educational research are less clear. Research designed solely for program or quality improvement is typically viewed as scholarship but not necessarily research. [6] It is hard to determine when such internally directed information gathering becomes publicly shared. And as Mavis and Henry have noted, "our informal observation is that many medical educators set out to conduct evaluations to improve educational programs but after the evaluation is complete, the possibility of publishing or presenting the findings emerges, clouding the intent of the original activity." [5] Our informal observation is exactly the same at our institution. This is complicated by a unionized environment in our institution wherein scholarship and research are rewarded beyond the rewards offered for simply improving teaching methodologies in the classroom or clinic, which is expected of all faculty.

While there is some literature in medical education which examines faculty understanding of institutional policies regarding human subjects research [7,8], there is none within the chiropractic profession. Using the search terms *chiropractic, institutional review boards, biomedical ethics, program development *and *curriculum development *in various combinations, we searched PubMed, MANTIS, CINAHL, and the Index to Chiropractic Literature, and found no papers addressing chiropractic faculty understanding of IRB procedures or policies. However, given the rising complexity of clinical and educational research in chiropractic, improving such understanding is not just desirable, it is mandatory. The purpose of this study was to provide a baseline from which training and development in the research process could be designed to assist faculty with scholarly endeavors. Specifically, we wanted to determine the level of understanding in the faculty of a single chiropractic institution about IRB policies in place at the institution, especially policies related to using student assessment and curriculum evaluation for scholarship. Our long term goal is to enhance the output of quality research from chiropractic institutions and prevent problems that may hamper the ability to share information gleaned from such research.

## Methods

An anonymous survey was distributed to all full-time faculty members at the Palmer College of Chiropractic Davenport Campus. In it, 4 scenarios were described addressing a student assessment activity or a curriculum evaluation activity. Participants were asked to indicate what they believe the correct decision was regarding whether that activity requires consideration by the IRB. They were able to choose from 3 possible courses of action: (1) submit an IRB application; (2) meet with the IRB chair or Human Protections Administrator; or (3) submit a conference abstract without IRB review. Using a template developed by Mavis and Henry [5], the survey also asked participants about: their knowledge of institutional policies related to IRB approval; their primary assignment at the college, either teaching, research, clinic or academic support; and, how many years of service they have served in the institution. The study was approved by the Palmer College of Chiropractic Institutional Review Board.

A meeting was held with both the head of the union and the president of the faculty senate to brief them on this project. A second meeting was held with the Vice President for Academic Affairs (VPAA) so that constituent areas of the college were informed and aware of the survey and its goals. A letter, drafted by the authors and agreed upon by the VPAA, the head of the union and the faculty senate president, was co-signed by all 3 and sent out to all full-time faculty, inviting their participation while at the same time noting the voluntary nature of that participation. A list of all full-time faculty members was obtained from the office of the VPAA.

Invitation letters and consent forms were mailed to 102 full-time faculty. Potential participants were invited to sign the consent form and return it through interoffice mail in a pre-addressed, sealed envelope. In lieu of witnessing the signature, when signed consent forms were received, one of us (MAH) sent an email to the participant faculty member asking them to confirm their consent to participate. After confirmation we sent the survey to participants in batches of at least 10 to preserve anonymity.

## Case Scenarios

Scenario 1: "Your department has responsibility for the on-going evaluation of the clinical skills curriculum for chiropractic students. In reviewing students' test scores from the course multiple-choice examinations and faculty performance ratings of students, you note some interesting relationships. In discussing these findings with the course director, you both agree they have educational significance and therefore you decide to submit an abstract to the next ACC-RAC meeting based on these data."

Scenario 2: "Your department has responsibility for the on-going evaluation of the chiropractic skills curriculum for chiropractic students. The chiropractic skills department chair inquired about the relationship between student performance in the second and third year of the chiropractic skills curriculum. Of particular interest is the bottom 20% of students based on faculty performance ratings and evaluations. To answer this question, second and third year students from the bottom 20% of the class were videotaped while demonstrating chiropractic clinical skills; these tapes were then reviewed by three faculty members, who rated the performance using standard checklists and rating scales. After reviewing the analyses of data, the department chair and faculty raters decided to submit an abstract to the next ACC-RAC conference."

Scenario 3: "You have been assigned by your department chair to gather information related to the ongoing programmatic accreditation process, and this requires you to work in the office of the registrar to look through blinded (names removed) computer records for the purpose of tabulating information related to entering grade point averages. This information will be included in the accreditation report. Once you have completed this project, your chair suggests that sharing the information with others at a conference might be a worthy undertaking."

Scenario 4: "You have developed an interest in an educational innovation, which you intend to try in your class. In doing so, you realize that you can use the results from your study as the means to apply for a Thelma Mack-Fordyce award, and as a result you decide to ask a colleague from outside your department, but who has a similar interest, to become involved with you in this project. Your results are coded by student matriculation number, and the results are promising. You therefore submit an application for the Thelma Mack-Fordyce award."

Data were entered by both authors into an Excel spreadsheet and verified for all 3 parts of the survey. All comments written in response to the open-ended question were entered, coded and reviewed for themes. This is a descriptive study and data are presented as frequencies for categorical items. No *a priori *hypotheses were planned for this initial survey.

## Results

Of the 102 full-time faculty members, 66 returned consent forms and confirmed their interest to participate via email, and 56 surveys were returned for a 55% response rate. Two weeks after the initial invitations were sent to faculty, only 48 consent forms and 29 surveys were returned. Follow-up invitations were sent via email at 2 and 4 weeks to 54 and 43 non-participants, respectively. At 8 weeks, an email was sent to all who consented to participate, thanking them for their participation and encouraging anyone who had not yet returned their survey to do so; this final communication yielded an additional 7 responders. The 55% return rate reflects all surveys returned after the third and final follow-up invitation to participate. All communications via email listed faculty member names in the blind carbon copy field.

By primary assignment, responses rates included 52% from teaching (25/48), 56% of clinics (23/41), 67% of eligible members of research, excluding the authors, (4/6), and 80% of academic support (4/5). Table 1 provides information on assignment and years of service.

**Table 1:** Survey respondent years of full-time service by primary college assignment

	Respondent Group				
	All Respondents	Teaching	Clinic	Research	Academic Support

Years of Service (n = 56)					
Less than 5	11	4	4	1	2
5–10	8	2	2	3	1
More than 10	37	19	17	0	1
**Totals:**	**56**	**25**	**23**	**4**	**4**

Table 2 lists the responses to case scenarios for all respondents and for respondents by primary assignment. As per college policy, for all scenarios, the correct answer was to talk with the IRB chair or the Human Protections Administrator. In no scenario did the level of correct answers by all respondents score higher than 41% (Scenario 1) and in most, the scores were closer to just under 1 in 3, with the 4^th ^scenario scoring lowest at 27%. Table 3 lists the responses to case scenarios for all respondents and for respondents by years of service. The years of service did not appear to influence selecting the correct response across all four scenarios.

**Table 2:** Responses to case scenarios by primary college assignment

	Response to Case Scenarios					
**Scenario 1:**	Submit IRB application		Talk with IRB chair or Human Subjects Administrator		Submit a conference abstract without IRB review	

All respondents (n = 56)	16	29%	23	41%	17	30%
Teaching (n = 25)	6	24%	11	44%	8	32%
Clinic (n = 23)	9	39%	7	30%	7	30%
Research (n = 4)	0	0%	2	50%	2	50%
Academic Support (n = 4)	1	25%	3	75%	0	0%
						
**Scenario 2:**						
All respondents (n = 56)	11	20%	22	39%	23	41%
Teaching (n = 25)	4	16%	11	44%	10	40%
Clinic (n = 23)	7	30%	9	39%	7	30%
Research (n = 4)	0	0%	1	25%	3	75%
Academic Support (n = 4)	0	0%	1	25%	3	75%
						
**Scenario 3:**						
All respondents (n = 55)	32	58%	16	29%	7	13%
Teaching (n = 24)	17	71%	5	21%	2	8%
Clinic (n = 23)	13	57%	8	35%	2	9%
Research (n = 4)	0	0%	2	50%	2	50%
Academic Support (n = 4)	2	50%	1	25%	1	25%
						
**Scenario 4:**						
All respondents (n = 55)	36	65%	15	27%	4	7%
Teaching (n = 25)	18	75%	5	21%	1	4%
Clinic (n = 23)	14	61%	7	30%	2	9%
Research (n = 4)	2	50%	1	25%	1	25%
Academic Support (n = 4)	2	50%	2	50%	0	0%

**Table 3:** Responses to case scenarios by years of full-time service

	Response to Case Scenarios					
**Scenario 1:**	Submit IRB application		Talk with IRB chair or Human Subjects Administrator		Submit a conference abstract without IRB review	

All respondents (n = 56)	16	29%	23	41%	17	30%
Less than 5 (n = 11)	2	18%	4	36%	5	45%
5–10 (n = 8)	2	25%	3	38%	3	38%
More than 10 (n = 37)	12	32%	16	43%	9	24%
						
**Scenario 2:**						
All respondents (n = 56)	11	20%	22	39%	23	41%
Less than 5 (n = 11)	2	18%	5	45%	4	36%
5–10 (n = 8)	0	0%	2	25%	6	75%
More than 10 (n = 37)	9	24%	15	41%	13	35%
						
**Scenario 3:**						
All respondents (n = 55)	32	58%	16	29%	7	13%
Less than 5 (n = 11)	7	64%	3	27%	1	9%
5–10 (n = 8)	2	25%	3	38%	3	38%
More than 10 (n = 37)	23	62%	10	27%	4	11%
						
**Scenario 4:**						
All respondents (n = 55)	36	65%	15	27%	4	7%
Less than 5 (n = 11)	8	73%	3	27%	0	0%
5–10 (n = 8)	4	50%	2	25%	2	25%
More than 10 (n = 37)	24	65%	10	27%	3	8%

The mixed response to case scenarios is supported by the knowledge level for college policies regarding educational research and for obtaining student consent. (Table 4) Sixty-five percent of respondents indicated they were unsure whether Palmer had any policies in place at all, while 4% felt that no such policies were in place. Just over one-quarter (27%) were correct in noting that students can decline consent, while more than half (54%) did not know whether there were any procedures governing student consent. Just 3 (5%) felt that matriculation was conditional on consent, not noting the coercive nature of such a comment, and another 8 (14%) felt that there were no policies at all regarding student consent to research. Just over two-thirds of all respondents indicated they have had discussions with others at the institution about IRB requirements for using evaluation data for faculty scholarship.

**Table 4:** Knowledge of institutional policies by primary college assignment

	Respondent Group									
	All Respondents		Teaching		Clinic		Research		Support	

Does Palmer College of Chiropractic have formal policies for the use of existing educational evaluation data for faculty scholarship? (n = 55)										
Yes	17	31%	8	32%	8	36%	1	25%	0	0%
No	2	4%	0	0%	1	5%	0	0%	1	25%
Unsure	36	65%	17	68%	13	59%	3	75%	3	75%
**Totals:**	**55**		**25**		**22**		**4**		**4**	
										
Which best describes procedures in place a Palmer College of Chiropractic for obtaining consent from students to use their performance data and test scores for educational research and scholarship? (n = 56)										
Students can decline consent	15	27%	7	28%	7	30%	0	0%	1	25%
Matriculation conditional on consent	3	5%	1	4%	0	0%	1	25%	1	25%
There are no procedures	8	14%	5	20%	3	13%	0	0%	0	0%
Don't know	30	54%	12	48%	13	57%	3	75%	2	50%
**Totals:**	**56**		**25**		**23**		**4**		**4**	
										
Have you participated in discussions with others in your institution about IRB requirements for using evaluation data for faculty scholarship?(*Mark all that apply*)										
Faculty in your department	23	27%	9	25%	12	32%	1	25%	1	17%
Faculty in other departments, not research	8	10%	4	11%	2	5%	1	25%	1	17%
Faculty in the research department	14	17%	7	19%	7	18%	0	0%	0	0%
College faculty meetings	1	1%	0	0%	0	0%	0	0%	1	17%
Dean, administrators, etc.	13	15%	5	14%	8	21%	0	0%	0	0%
No discussion reported	22	26%	9	25%	8	21%	2	50%	3	50%
Other	3	4%	2	6%	1	3%	0	0%	0	0%
**Totals:**	**84**		**36**		**38**		**4**		**6**	

There were 11 responses in the space provided on the final page of the survey to comment about any of the survey questions or scenarios. The responses mainly indicated a lack of knowledge related to IRB policies and procedures. "Many faculty are interested in being involved in research projects. Most lack knowledge on how to start, who on campus could give them some guidance, or why an IRB is necessary." And, "I have recently come to realize (that) the expectations of submitting applications to the IRB for approval. The scenarios (some of them) appear to be too late to submit to the IRB, so I guess I still need a better understanding of this process." And finally again, "My answers are guesses. I have no knowledge about IRB procedures and policies" and "To be totally honest, I am not 100% sure what an IRB is." The indication here is that there is a sincere interest in conducting research but a lack of knowledge as to the processes, policies and procedures necessary to successfully do so. It is interesting to note that Palmer College has a well-staffed research department, the largest the chiropractic profession has ever seen. Between the 3 campuses there are 15 faculty members devoted to research, and more than 40 staff. There is a perception among faculty that it is the responsibility of the research department to do research; they are to teach or to offer clinical care, depending on appointment. The scholarship aspect often is lost under the needs of teaching, whether in classroom or clinic.

Other responses offered clarification of answers, ie, "In scenario 4, I would have thought that an IRB application would need to be submitted before the students were videotaped, since only a subset of the students were involved." And, "I do not believe that Mat #s (Author's note: matriculation number) can be published & I would think that this meeting would establish a code by which the info could be published." Also, one comment offered clarification for each scenario answer: "Scenario 1: Answer reflects not knowing if student info was anonymous. Scenario 2: Answer reflects anonymity. Scenario 3: Reflects that students can be traced via matric. #. Scenario 4: Reflects lack of anonymity due to videotape." These comments indicate confusion about institutional policies for educational research and correlate with the lack of knowledge shown in the survey results.

## Discussion

It is important to note that Palmer College has a newly unionized faculty, which creates difficulty in coordinating activities across the campus. Faculty are assigned roles through union leadership, are required to commit a certain number of hours per week for service activities, but generally shy away from those that they feel will not be counted as service because additional service may lead to higher pay. Given this, an approach that involved positive support from all sides of the faculty would lead to better results; thus, a letter was directed to all faculty from the VPAA, the head of the union (which is associated with the American union AFSCME), and the Faculty Senate President.

At the Palmer College of Chiropractic, which constitutes 3 campuses located in 3 separate states and operating as 1 institution, college policy mandates that all research proposals must first be submitted to the Human Protections Administrator (HPA). The HPA makes a determination as to whether the proposed research is exempt from consideration from the IRB. If the answer is "yes," the faculty member is then free to conduct his or her study; if "no," then the proposal must be submitted to the IRB for its consideration. At the IRB level, the decision may be made to expedite the review (so that the decision to approve may be made solely by the IRB chair or designee) or to have the proposal undergo full review.

Given this policy, the correct answer to all 4 scenarios is the same. In all cases, faculty are to submit the proposal to the HPA; in this survey, answer 2 is correct in that the faculty has to meet first with the IRB chair (who will refer them to the HPA) or the HPA him or herself. The survey is worded to focus on course of action, not ultimate disposition of the proposal. In some scenarios, the ultimate decision may be to exempt the proposal (thus, ending the scenario at the point of submission to the HPA) and in others it may be necessary to have the IRB ultimately review the proposal. This study focused on faculty understanding of the policies that are in place. While many faculty members may assume that all research requires a complete IRB application, this is not the case, and there is no reason for faculty to prepare full IRB applications for research that is exempt. Tables 2 and 3 shows the frequency counts of responses from each question. Given this, results are middling at best.

In scenarios 1 and 2, approximately 60% answered they would submit an IRB application or submit a conference abstract without IRB review (scenario 1, 30% and 29%; scenario 2: 41% and 20%). This suggests that they believe the research as described is institutional in nature, has no confidentiality issues, and is thereby exempt- forgetting that they cannot make that decision on their own. In scenarios 3 and 4, more of those who answered incorrectly now believe that they should submit to the IRB rather than submit a conference abstract without HPA review. (Tables 2 and 3) This suggests that the increasing complexity of the research, as described, raises questions particularly regarding confidentiality, since in these 2 scenarios issues of matriculation number and videotape appear. The ethical issue of generalized knowledge remains the same in all 4 scenarios, however; it is this issue that requires the HPA consideration, among other important issues.

In the original work of Mavis and Henry [5] upon which the current survey is based, their primary concern was that all too often medical educators set out to conduct classroom evaluations with the goal of improving educational programs, but later decide the information they have gathered may be beneficial to share with others via publication or presentation at a conference. That same issue has occurred at Palmer College (and, we believe, occurs at other chiropractic institutions), where the past Human Protections Administrators were often confronted with research such as a classroom survey that a faculty member then wished to use as a conference submission later. And all too often, either a retroactive approval was provided or in some cases a denial was made. With the new union and with a need to enhance scholarship, tension arises among the faculty. They wish to be involved in scholarship but often lack the skills to conduct rigorous quantitative research; however, educational research is often a means to begin the process of research socialization. And the ethics involved becomes critically important as a result.

Mavis and Henry [5] surveyed medical educators in 2 types of institutions: research-based and community-based. They used 2 case scenarios to guide the survey. Their response rate was close to 90%, as they were able to conduct much of the survey while in direct contact with the educators at a conference. 121 individuals participated. In both scenarios, the correct answer was that IRB contact was required; results demonstrated that 29% agreed with this in case 1 and 47% in case 2; a slightly higher number felt that a discussion with the IRB chair would suffice for case 1, while a slightly lower number answered that way for case 2. Nearly one-third felt that a conference presentation could be made with IRB review in case 1, while 16% felt that same in case 2. More than half the people in the study were unaware if their institution had any policies in place regarding educational research. Our results are equally startling in this regard. What is even more troubling is that 2–3 members of the research department, recognizing that only 4 responded, answered incorrectly for all scenarios.

Matot et al note similarly troubling findings [9]. Over a one-year period for all publications in *Critical Care Medicine*, nearly one-quarter of articles (24%) were found to not be reviewed by IRBs, and from these 27% were retrospective, 21% involved the use of standard therapy, and 18% used invasive procedures. For studies that did have IRB approval, 20% did not report obtaining informed consent. While some of this, notably the retrospective studies, may be exempt from the need for IRB approval, certainly not all is. In a commentary on Matot's article, Danis and Grady [10] note that efforts to allow those receiving standard care in randomized trials not give consent have not been supported as yet. They also note the responsibility editors have in clarifying this issue.

Roberts et al [8] raise certain concerns with regard to educational research. They state that (1) there is a critical moral difference between education research and scholarly education practice; (2) education research is governed by federal regulations requiring more rigorous safeguards than often exist in usual education practice; and (3) it is increasingly being recognized that students who participate in research have several characteristics in common with members of special populations. General faculty may not be sensitive to these risks. In looking at how well medical faculty appropriately disclosed the ethics of their educational research, Roberts et al [8] found that the level of compliance was very low, with nearly half of the papers providing no information at all about ethical safeguards. And this did not change over time, even as the issue became more public. The two critical issues for Roberts are the inherent role conflicts between teaching and educational research for an instructor, and the safeguard and compliance issues that must be addressed. Educational institutions must be aware of these tensions.

Mayeda and Takase [11] note that better ethics training helped to decrease the frequency of lawsuits. The efforts they made in surveying postgraduate trainees has implications for training postgraduate fellows in clinical research as well as faculty involved in research. Fostering awareness of issues was found, at least in their Japanese population, both useful and well accepted.

The results indicate that across the spectrum, faculty members at Palmer College do not always understand when it is necessary to seek IRB approval, seek consideration by the Human Protections administrator, do not understand the appropriate protocols for human subject approval, and are unaware of institutional policies regarding educational research. Further, many never engage another faculty member for information. These results will inform interventions and training to rectify this problem.

### Lessons Learned from this Pilot Study

Despite several reminder letters, our 55% response rate was disappointing, given we were surveying an audience with which we had close and easy contact. In addition, we found that there were 10 people who signed consent forms indicating their willingness to participate in the project, and who then did not return their completed survey. Because this was an anonymous survey, we have no way of identifying non-participants, nor their reasons for non-participation. The vast majority of participants responded to the first request for consent and also returned their surveys promptly. Two weeks after we mailed out the consent forms, we received 48 completed forms along with 29 completed surveys. At that point, a reminder was mailed out that included another copy of the consent form, and within 11 days from that time, we had received an additional 12 signed consent forms and 8 completed surveys. Despite several further reminders, the remaining signed forms and surveys trickled in over the duration of the project.

We intend to broaden the scope of this survey for chiropractic colleges internationally. Challenges to using a mail survey are many, and include obtaining complete faculty lists for all the chiropractic colleges, maintaining continual contact so that response rates are optimized, and the costs of sending out regular reminders. There are several potential solutions. One solution is to develop a web-based survey for ease of administration and communication. A second would be to use representative sampling from audiences captured at various chiropractic scientific symposia. It is desirable to have higher than a 55% response rate, and yet obtaining that even at a single institution took substantial time and energy.

Another concern relates to anonymity and confidentiality. Though we assured all faculty, via letter, that their involvement was anonymous and confidential and that they were free to not participate, comments were made by at least 2 respondents about fears that the use of email and blind copying would still allow the institution to determine that they were involved. In an adversarial situation, such as occurs in unionized environments, trust may at times be lacking and fear of reprisal may lead participants to opt out and not be involved at all, even for a survey such as this which has no political immediacy or import. It would take significant energy, time and will to obtain specific computer information about who did or did not participate, though this did not deter 2 participants from noting that concern. This concern will need to be addressed with a web-based survey.

This pilot survey was tailored to examine the understanding of Palmer faculty about IRB policies. A broader survey will have to consider the nature of the response choices for the scenarios, given that some chiropractic colleges may not have assigned Human Protections Administrators or that some nations may have differing legal requirements concerning consent and human subject research.

### Limitations

This is a pilot study and surveyed a relatively small number of respondents. Given this, we were able to obtain stable response rates across all 4 assignment areas. Because the survey was designed to be anonymous, we can say little about the respondents other than that a greater number of them came from those with more years of service in the institution. It is unclear whether gathering more demographic information about individual respondents is of value and, because of the small number of faculty in some departments, this may unmask the anonymity of respondents. We believe all faculty should understand their own institutions' IRB policies regardless of demographics.

## Conclusion

The results here are in harmony with those of Mavis and Henry [5]. Palmer faculty have only modest understanding about institutional policies regarding the IRB and human subject research, especially as it pertains to educational research. Given this, the institution needs to develop methods to provide knowledge and training to faculty. This has already been initiated; a 1-hour program on basic knowledge of what constitutes human subject research and which provides an overview of Palmer policies was taught at a faculty in-service in October of 2006. In addition, information has been placed on the Palmer Center for Chiropractic Research website that instructs faculty on the basics of project development, IRB submission, Human Protections issues, exemption and research ethics. It is likely that other colleges in the chiropractic profession face the same challenge, and a larger survey will provide these data.

## Competing interests

Neither author has any competing interests to declare. Dr. Lawrence is the Human Protections Administrator for Palmer College of Chiropractic.

## Authors' contributions

DJL came up with the initial conception and design for the study, co-wrote the manuscript and researched the literature. MAH coordinated the survey, refined and clarified the methodology, and co-wrote the manuscript. Both authors read and approved the final manuscript.
